# Diagnostic performance of treadmill exercise cardiac magnetic resonance: the prospective, multicenter EXACT trial

**DOI:** 10.1186/1532-429X-18-S1-O132

**Published:** 2016-01-27

**Authors:** Subha V Raman, Jennifer A Dickerson, Wojciech Mazur, Timothy C Wong, Erik B Schelbert, Debbie Scandling, Jason Craft, James Min, Cheryl Bartone, Ernest L Mazzaferri, Paaladinesh Thavendiranathan, John Arnold, Robert Gilkeson, Orlando P Simonetti

**Affiliations:** 1 Ohio State University, Columbus, OH USA; 2 The Christ Hospital, Cincinnati, OH USA; 3 University of Pittsburgh, Pittsburgh, PA USA; 4 Weill-Cornell Medical College, New York, NY USA; 5 Case Western Reserve, Cleveland, OH USA

## Background

Stress cardiac magnetic resonance (CMR) typically involves pharmacologic agents that do not replicate symptoms and signs during typical exertion. Recent studies suggest excellent diagnostic performance of pharmacologic stress perfusion CMR compared to nuclear scintigraphy (SPECT); however, comparison of treadmill exercise stress CMR to SPECT has not been done.

## Methods

Patients clinically referred for treadmill stress SPECT for the evaluation of known or suspected CAD were prospectively enrolled across 4 centers**.** After rest Tc99m SPECT imaging, patients underwent resting cine CMR. In-room stress was then performed using an MR-compatible treadmill with continuous 12-lead ECG monitoring and the Bruce exercise protocol. At peak stress, Tc99m was injected and patients were rapidly returned to their prior position in the magnet for real-time, free-breathing post-exercise cine and perfusion CMR. Following recovery monitoring with the table brought outside of the magnet bore, recovery cine and rest perfusion followed by late gadolinium enhancement acquisitions concluded the CMR portion of the exam. Stress SPECT images were then acquired in the adjacent nuclear laboratory. Patients not referred for invasive coronary angiography (ICA) within 2 weeks of stress imaging underwent coronary angiography with computed tomography (CTA). Diagnostic accuracy and prognostic value of treadmill stress CMR vs. SPECT were evaluated.

## Results

Of 210 patients (age 57 ± 11 years, 40% female) completing the study protocol, exercise time averaged 9.0 ± 2.6 min of the Bruce treadmill protocol achieving 10.2 ± 3.0 METS and reaching 97 ± 11% of the age-predicted maximum heart rate. After exercise termination, stress cine imaging was completed within 47.7 ± 16.7 sec and stress perfusion by 91.0 ± 33.6 sec. Compared to angiography, exercise CMR demonstrated sensitivity of 86%, specificity of 100%, positive predictive value of 100% and negative predictive value of 98%. There was strong agreement between treadmill stress CMR and angiography (κ = 0.91), but only moderate agreement between stress SPECT and angiography (κ = 0.55) and between CMR and SPECT (κ = 0.48). At 12-month follow-up, no major adverse cardiac events had occurred in this cohort of ambulatory patients referred for stress imaging.

## Conclusions

The multicenter EXACT trial indicates both diagnostic and prognostic value of treadmill stress CMR in typical patients referred for exercise SPECT.

**Figure 1:**
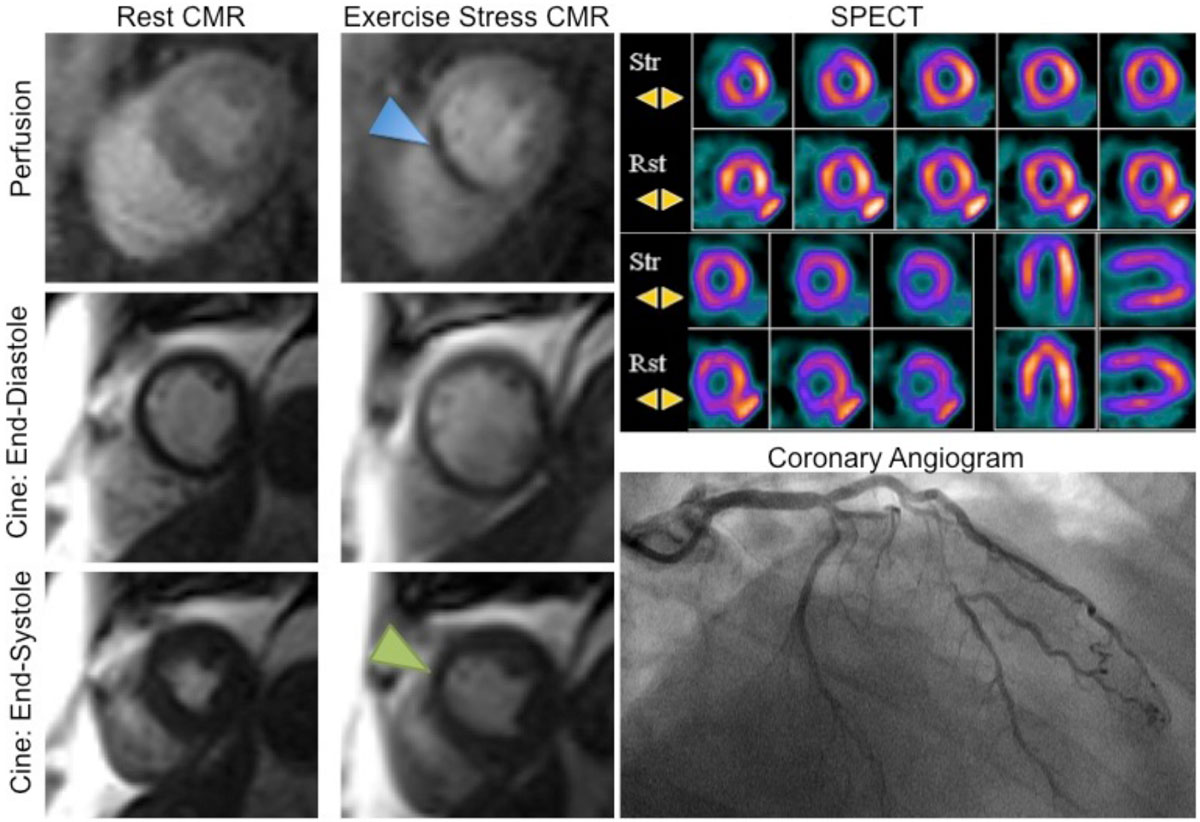
**A 59 year-old male referred for exercise SPECT underwent the hybrid treadmill stress CMR-SPECT protocol**. While SPECT (upper right color image) suggested a perfusion abnormality, cine and perfusion CMR clearly showed both wall motion and perfusion abnormalities present only on stress images (blue and yellow arrowheads, respectively), with viable myocardium by LGE (not shown). Invasive angiography (lower right) confirmed high-grade left coronary artery disease.

